# Geographic variation in thermal tolerance of western corn rootworm

**DOI:** 10.1038/s41598-025-08768-8

**Published:** 2025-07-18

**Authors:** Karl A. Roeder, Skylar Drey, Jesse D. Daniels, Jamieson C. Botsch

**Affiliations:** 1 USDA, Agricultural Research Service, North Central Agricultural Research Laboratory, 2923 Medary Ave, Brookings, SD 57006 USA; 2 Department of Biology, Austin Peay State University, Clarksville, TN 37044 USA; 3 Oak Ridge Associated Universities, Oak Ridge, TN 37831 USA

**Keywords:** Chill coma recovery, CT_max_, CT_min_, Knock-down resistance, Temperature, Agroecology, Ecophysiology, Ecology, Physiology, Zoology, Entomology, Ecology, Agroecology, Ecophysiology

## Abstract

Western corn rootworm, *Diabrotica virgifera virgifera*, is one of the most economically important crop pests in the world with estimates of damage and control costing over $1 billion USD annually. Yet despite an abundance of research devoted to studying rootworm biology in the central Corn Belt of the United States, information on key aspects of their thermal biology is still lacking. In this study, we quantified thermal metrics of western corn rootworm populations from across their range in the United States: we measured critical thermal limits, knock-down resistance, and chill coma recovery for male and female rootworm from 13 laboratory colonies that were collected across 1985 km at locations that varied by up to 5.7 °C in mean annual temperature. We further use these data to test a model from thermal ecology—the thermal adaptation hypothesis—which posits that (1) thermal limits track environmental temperatures and (2) more thermally variable environments support organisms with broader thermal ranges. In doing so, we found that thermal traits varied across populations. However, only heat tolerance traits (critical thermal maximum and knock-down resistance) tracked historical averages of mean annual temperature. Rootworm originating from more thermally variable environments did not exhibit broader thermal ranges. While theory often predicts cold tolerance should track environmental temperatures, our results suggest this pattern may disappear if organisms are reared in the laboratory for multiple generations and instead a legacy effect may exist for heat tolerance that is rarely reported.

## Introduction

For ectothermic insects, one of the most important abiotic variables is undoubtedly temperature as it governs—amongst many other things—activity, phenology, development, metabolism, survival, and reproduction [1–3]. Moreover, temperature can limit the spatial and temporal distribution of economically important species, which are often constrained by their thermal tolerances [4], and further impact the amount of insect-mediated ecosystem services being provided to natural and managed ecosystems. For example, a 5 °C increase in temperature during the flowering period of *Borago officinalis* negatively affected visual floral traits and floral rewards, resulting in a fourfold decrease in foraging activity by the bumble bee, *Bombus terrestris* [5]. Alternatively, certain pest species like the coffee berry borer, *Hypothenemus hampei*, are already benefiting from warmer temperatures as predictive models suggest that even slight increases in temperature could result in further altitudinal shifts that will enable range expansions into production areas of high value *Coffea arabica* [6]. How insect species respond to temperature thus remains an ecologically broad and complex question with potential implications for global economics and food production [7].

To better understand how insects are affected by temperature, thermal biologists have often focused on physiological traits like critical thermal limits (CT_max_ – critical thermal maximum; CT_min_ – critical thermal minimum), knock-down resistance, and chill coma recovery [8, 9]. Each of these traits represent different ways in which insects can tolerate and even recover from temperatures near their physiological limits, resulting in numerous primary studies and syntheses for ants, bees, beetles, dragonflies, flies, grasshoppers, mayflies, termites, true bugs, and other insect taxa [4, 10–19]. Yet despite this ever-growing body of literature, data is often sparse on how thermal traits vary within species (i.e. intraspecific variation), especially from populations across large geographic areas. Intraspecific trait variation has continued to be identified as an important component in population and community dynamics as traits are more regularly recognized to be plastic [20–22]. Thus, if we are to accurately forecast the future spatial extent of ecologically important insects, inclusion of intraspecific variation in traits from different populations will be key.

Western corn rootworm, *Diabrotica virgifera virgifera* (hereafter WCR), is one species that may be useful for studying intraspecific trait variation in thermal tolerance as they are a geographically widespread, economically important pest of corn (*Zea mays* L.) with annual estimates of damage surpassing $1 billion USD [23–25]. WCR has continually expanded their range over the past century, advancing from the central Great Plains to the East Coast of the United States by the mid-1980s [25]. A decade later, WCR was introduced into Europe and now occupies over 20 countries [26–28]. To inhabit such variable environments, rootworms have remained opportunistic and flexible, maintaining high genetic diversity within populations [29] including variants that disperse into non-corn fields to bypass management strategies that employ crop rotation with soybean [*Glycine max* (L.)] [30]. Such genetic diversity could have implications for thermal traits as components of heat tolerance may be heritable and/or constrained [3, 31, 32]. Cold tolerance often tracks environmental temperatures though and is more variable across geography [33–35]. However, no studies to date have examined if geographic variation in rootworm thermal traits exists and if such intraspecific variation matches the above-mentioned theoretical predictions from thermal ecology.

Here we quantify thermal metrics of WCR populations from across their range in the United States. We measured critical thermal limits, knock-down resistance, and chill coma recovery for male and female rootworms whose populations originate from 13 locations that span more than 1985 km and vary by up to 5.7 °C in mean annual temperature. We further use these data to test a model from thermal ecology—the thermal adaptation hypothesis—which posits that (1) thermal limits track environmental temperatures and (2) more thermally variable environments support organisms with broader thermal ranges [35–37].

## Materials and methods

### Geographic populations

**Fig. 1:**
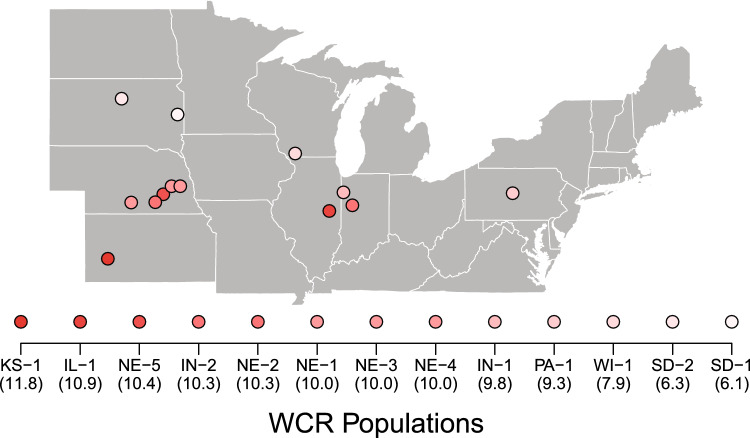
Geographic distribution of the 13 tested western corn rootworm (WCR) populations from across the United States. Points are color coded using a gradient from dark red (hottest mean annual temperature in Kansas) to light pink (coolest mean annual temperature in South Dakota). Code names (e.g. KS-1) correspond to colony locations described in Table S1 with mean annual temperatures in °C (BIO1 in WorldClim) listed below each population.

Thirteen geographically distinct WCR populations have been maintained at the USDA-ARS North Central Agricultural Research Laboratory in Brookings, SD for ca. 30 years. Original field collections occurred between 1994 to 2013 in Illinois, Indiana, Kansas, Nebraska, Pennsylvania, South Dakota, and Wisconsin (Fig. 1, see also Table S1 for more information). Detailed rearing methods are similar across populations [38–40]. For all thermal trials, we used an equal number of female and male adults that were collected three days after eclosion. Additional rootworms were also kept at ambient room temperature (22 °C) for each thermal tolerance trial as a control—all of which survived.

WorldClim values for mean annual temperature (i.e. bioclimatic variable BIO1) and annual temperature range (BIO7) averaged from 1970 to 2000 were extracted using the latitude and longitude for the centroid of the county in which each population was collected. Other bioclimatic variables like mean temperature of the warmest quarter (BIO10) and mean temperature of the coldest quarter (BIO11) were considered, as they represent extreme temperatures that rootworms experience, however they were multicollinear with mean annual temperature (Variance Inflation Factor > 3) and not analyzed further. The spatial resolution was ca. 18.5 km × 18.5 km (10 arcmin).

### Critical thermal limits

Critical thermal maxima (CT_max_) and critical thermal minima (CT_min_) were measured using a dynamic heating/cooling ramping assay that has been commonly used to measure thermal tolerance [9, 19, 41–43]. For each population and for each assay, we used 30 new individuals. Assays were conducted by placing individual rootworm into 1.5 ml microcentrifuge tubes that had been modified with cotton to remove a thermal refuge in the cap. Microcentrifuge tubes were then placed into a prewarmed/precooled EchoThermTM IC20 heating/chilling dry bath (Torrey Pines Scientific, Carlsbad, CA, USA) set at 15 °C for CT_min_ and 35 °C for CT_max_. After an assay began, we checked rootworms every 10 min to see if they had reached their critical thermal limit by rotating the vials and looking for a righting response. Dry bath temperature was then increased/decreased 1 °C (ramping rate = 0.1 °C min^-1^) and the process was repeated until all rootworms had lost muscle control. Starting temperatures and ramping rates were chosen based on preliminary work [44].

### Knock-down resistance and chill coma recovery

We used a new set of 30 individuals from each geographic population to determine how long individuals could survive at temperatures near their critical thermal maxima (i.e. knock-down resistance). We did this by placing individual rootworm into 1.5 ml microcentrifuge tubes in a dry bath (Torrey Pines Scientific, Carlsbad, CA, USA) set at 41 °C—a value close to their CT_max_ (previous CT_max_ trials in this study and [44]). We then checked rootworms every 10 min for 120 min to determine if and at what time individuals could no longer right themselves.

We used 0 °C—a value close to the lowest CT_min_ for WCR (previous CT_min_ trials in this study and [44])—to approximate the temperature at which individuals enter a reversible, paralyzed state known as a chill coma. Using a new set of 30 individuals from each population, we quantified the length of time it took to recover from a chill coma (i.e. chill coma recovery) by first placing individual rootworm into 1.5 ml microcentrifuge tubes in a dry bath (Torrey Pines Scientific, Carlsbad, CA, USA) set at 0 °C for 2 h. We then removed rootworms from the dry bath after the elapsed time and checked individuals every 5 s to see if they could right themselves within a two-hour observation period at ambient room temperature (22 °C).

### Statistical analyses

All analyses were run in R, version 4.0.1 [45]. We used generalized linear models (GLMs) with gaussian error distributions to compare critical thermal values (CT_max_ and CT_min_) of WCR across populations. In all models, we included sex (male/female) as a main effect. Pairwise contrasts among populations were performed using the ‘emmeans’ package [46]. Knock-down resistance and chill coma recovery for different populations were compared using the survdiff function, a log-rank test to compare two or more survival curves, in the “survival” package [47]. Survival curves were visualized using Kaplan–Meier plots and Restricted Mean Survival Times (RMSTs), which can be interpreted as the average survival time during a defined time period, were calculated for knock-down resistance and chill coma recovery.

The thermal adaptation hypothesis was tested by calculating population averages for each thermal trait (CT_max_, CT_min_, knock-down resistance, and chill coma recovery) and comparing those averages to mean annual temperature (BIO1) from the centroid of the county in which each population was collected using ordinary least squares (OLS) regression. Similarly, to test if more thermally variable environments support organisms with broader thermal ranges, population level CT_range_s (i.e. CT_max_—CT_min_) were calculated and regressed against the annual temperature range of each site (BIO7). We refrained from calculating a similar metric between knock-down resistance and chill coma recovery as they represent different aspects of rootworm thermal biology (i.e. muscle failure compared to recovery). Pearson product-moment correlation coefficients were then calculated between each trait to look for positive or negative relationships.

## Results

### Critical thermal limits

**Fig. 2:**
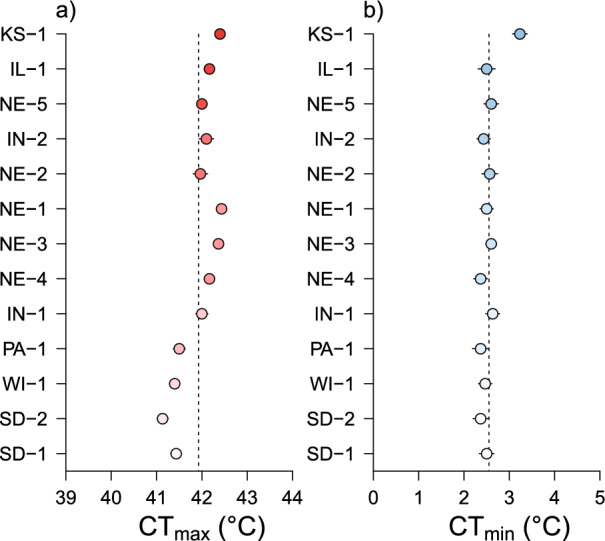
Critical thermal maxima (panel a; CT_max_) and minima (panel b; CT_min_) for 13 western corn rootworm (WCR) populations. Code names (e.g. KS-1) correspond to colony locations described in Table S1 and are arranged vertically from the hottest (dark red/blue) to coolest (light pink/blue) mean annual temperature. Vertical dashed lines represent the global mean for each trait with points being population means (± SE).

In total, we measured critical thermal limits for 780 WCR. The average CT_max_ across all individuals was 41.9 °C (range = 40 to 44 °C), while the average CT_min_ was 2.5 °C (range = 1 to 6 °C). Critical thermal limits varied across populations (Fig. 2) with individuals from Kansas ($\overline{X}$ = 42.4 °C) and Nebraska ($\overline{X}$ = 42.4 °C) having the highest heat tolerance and individuals from South Dakota ($\overline{X}$ = 41.1 °C) and Wisconsin ($\overline{X}$ = 41.4 °C) having the lowest (χ^2^ = 139.94, df = 12, *P* < 0.001). While differences were small, females were slightly more heat tolerant than males (+ 0.16 °C; χ^2^ = 5.66, df = 1, *P* = 0.017). Cold tolerance varied less with only the Kansas population maintaining an average CT_min_ above 3 °C (χ^2^ = 23.33, df = 12, *P* = 0.025). No statistical difference was detected in CT_min_ between sexes (χ^2^ = 2.95, df = 1, *P* = 0.086).

### Knock-down resistance and chill coma recovery

**Fig. 3:**
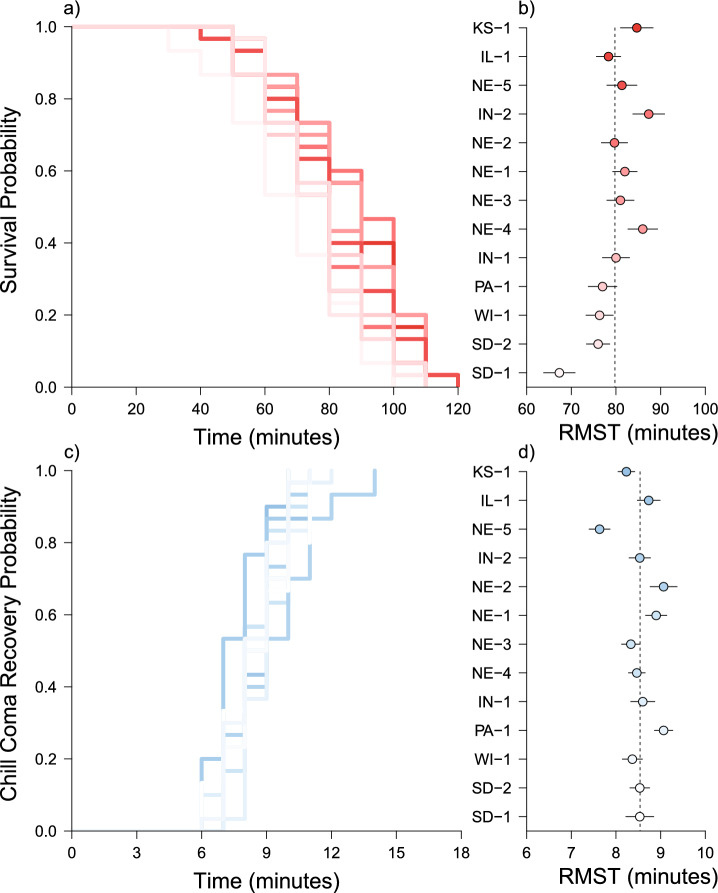
Knock-down resistance (panel a, b) and chill coma recovery (panel c, d) for 13 western corn rootworm (WCR) populations. Survival curves are visualized using Kaplan–Meier plots (panels a, c) with corresponding Restricted Mean Survival Times (RMSTs; panels b, d). Code names (e.g. KS-1) correspond to colony locations described in Table S1 and are arranged vertically from the hottest (dark red/blue) to coolest (light pink/blue) mean annual temperature. Vertical dashed lines in panels **b** and **d** represent the global mean for each trait with points being population means (± SE).

We measured 390 WCR for knock-down resistance and 390 WCR for chill coma recovery. Knock-down time varied across populations (Fig. 3a; χ^2^ = 33.1, df = 12, *P* < 0.001) with Restricted Mean Survival Times (RMSTs) ranging from a high in Indiana of 87.3 min to a low in South Dakota of 67.3 min (Fig. 3b). Sex was not a significant predictor of knock-down resistance (χ^2^ = 1.3, df = 1, *P* = 0.200).

All rootworms survived the chill coma recovery trial and were able to right themselves on average within nine minutes. The speed of recovery varied across populations (Fig. 3c; χ^2^ = 42.1, df = 12, *P* < 0.001) as RMSTs ranged from 7.6 to 9.1 min with the fastest and slowest times both occurring in populations from Nebraska (Fig. 3d). However, the magnitude of time difference for chill coma recovery across populations, while significant, was less than the difference observed for knock-down resistance. That is to say, knock-down RMSTs varied by up to 20 min across populations (Fig. 3b) while RMSTs for chill coma recovery varied by only 1.5 min (Fig. 3d). Sex was a significant predictor of chill coma recovery with females recovering 0.7 min faster than males (χ^2^ = 23.1, df = 1, *P* < 0.001).

### Geographic correlates of thermal traits

**Fig. 4:**
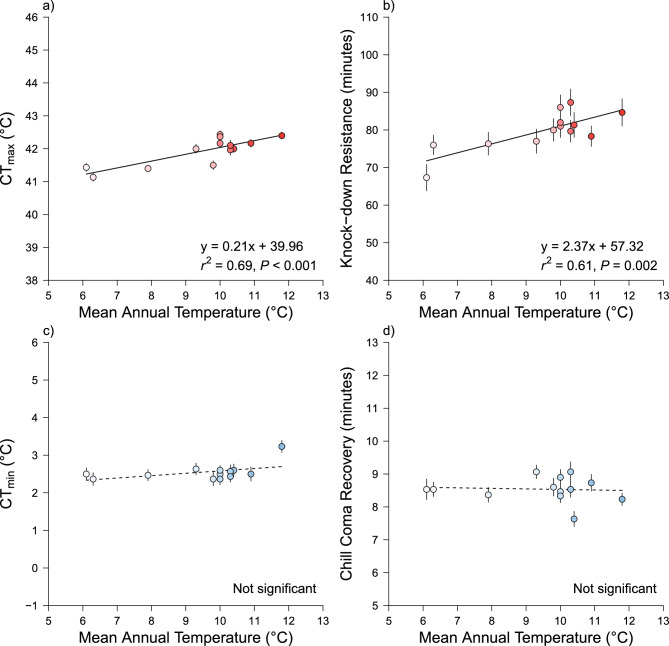
Geographic correlates of thermal traits for critical thermal maxima (panel a; CT_max_), knock-down resistance (panel b), critical thermal minima (panel c; CT_min_), and chill coma recovery (panel d). Points are populations means (± SE) regressed against mean annual temperatures in °C from the population origin (BIO1 in WorldClim).

WorldClim values for the origin of each population varied by 5.7 °C for annual mean temperature (BIO1: Range = 6.1 to 11.8 °C) and 10.9 °C for annual temperature range (BIO7: Range = 36.6 to 47.5 °C). WCR heat tolerance increased with mean annual temperature in a positive manner for both CT_max_ (Fig. 4a; y = 0.21x + 39.96, *r*^2^ = 0.69, *P* < 0.001) and knock-down resistance (Fig. 4b; y = 2.37x + 57.32, *r*^2^ = 0.61, *P* = 0.002). However, similar relationships were not observed for CT_min_ (Fig. 4c; *P* = 0.098) or for chill coma recovery (Fig. 4d; *P* = 0.813). Consequently, only CT_max_ and knock-down resistance were significantly correlated (t = 3.202, df = 11, *r* = 0.69, *P* = 0.008) and no other traits demonstrated trade-offs or constraints with each other (Table S2). More thermally variable environments also did not correlate with broader thermal ranges (*P* = 0.137).

## Discussion

Intraspecific trait variation in thermal tolerance of WCR did not follow theoretical predictions from thermal ecology, or many of the prior results from empirical studies. While variation occurred across populations for almost every trait, both metrics of heat tolerance—CT_max_ and knock-down resistance—were positively correlated with historical average temperatures, but cold tolerance was not. Moreover, more thermally variable environments in South Dakota, compared to less variable climates in Pennsylvania, did not support a broader range of thermal limits. Thus, we found limited support for the thermal adaptation hypothesis when looking across our populations of WCR.

### Population level differences

WCR currently occupy a rather large amount of geographic area in both the United States and parts of Europe as their distribution seems to be only restricted by the presence of corn and the abiotic conditions in which their host plant is grown [25, 26]. By examining thermal traits of WCR from across the United States, we sought to understand if intraspecific trait variation occurred in this species and if that variation may have aided their expansion to novel locations for corn production. Interestingly, differences were observed across populations for each thermal trait, but not in ways that we predicted.

One possibility for such a result is that local adaptation (i.e. genetic differentiation) among populations could be causing divergence in heat tolerance traits [20]. Thus, despite populations of WCR being reared in the laboratory under similar conditions for decades, genetic factors could be regulating heat traits to parallel the environmental conditions of the locations from whence they were collected—a legacy effect. Intraspecific patterns in heat tolerance have been observed in laboratory populations for other species like *Drosophila melanogaster*, in which differences in knock-down resistance across latitudes remained after 6 generations [48]. Yet, prior genetic research on WCR has found little differences across populations from Texas to New York with most of the reported genetic diversity simply occurring among individuals within a population [29, 49, 50]. It is unclear if any genes regulating heat tolerance were examined in these prior studies. However, one interesting result from our measured heat tolerance traits is that CT_max_ and knock-down resistance were the only two thermal metrics that were correlated (Table S2). Recent work has highlighted this link in other taxa like *Drosophila* where static and dynamic assays can provide similar information about heat tolerance and be mathematically modelled [51].

Cold tolerance traits like CT_min_, on the other hand, were similar across populations with only WCR from Kansas being statistically different and maintaining a worse tolerance compared to the other measured populations (Fig. 2). Chill coma recovery was more variable, yet still did not follow patterns predicted from theory. This was a bit surprising as prior research has often indicated that cold tolerance should more accurately track environmental conditions—a pattern observed in a variety of taxa like ants and flies [35, 52]. Thus, our initial prediction was that cold tolerance, and not heat tolerance, would be more likely to support the first part of the thermal adaptation hypothesis (i.e. thermal limits track environmental temperatures). One working hypothesis for this discrepancy is that cold tolerance is more plastic than heat tolerance, supported by observations that rearing conditions and temperatures that adults experience have both been shown to be important [53, 54]. Some have further argued that such environmental conditions may be more important than genetic differences for cold tolerance traits [20, 55]. Given that our rootworm populations have been reared at a constant 25 °C for multiple generations, there may not have been any selective pressure or reason for WCR to maintain colder thermal limits that parallel conditions from their original collection location. An alternative, but not mutually exclusive, hypothesis is that WCR is an unvoltine species with adults that are active for only a relatively short period of time during the summer. Thus, the likelihood of adult WCR experiencing temperatures that are low enough to affect cold tolerance traits would be quite minimal.

### Potential avenues of future research

One interesting discovery from this work is that differences between sexes were uncovered for each thermal metric. In every case, females performed better than males on average: CT_max_ was 0.16 °C higher, CT_min_ was 0.16 °C degrees lower, knock-down resistance was 1.65 min longer, and chill coma recovery was 0.7 min faster. One possible explanation is that female WCR are often larger than males by  ~ 0.06 mg (Range: Males = 1.4–5.9 mg, Females = 1.6–7.1 mg; dry mass values collected from a subset of measured individuals in this study). Yet, body size has a complicated relationship with insect thermal tolerance, resulting in a range of possible intraspecific relationships that can be positive, negative, or non-significant [15, 56, 57]. For WCR, much of the difference in weight occurs in the abdomen where female insects generally maintain a larger fat reservoir that is used for growth, metabolism, and reproduction [58–60]. Lipid levels have not been directly linked to thermal traits in many insects, but they could provide an energy reserve that can be mobilized to offset stressful temperatures [61–63]. If sex specific differences in thermal tolerance occur for other species, then accounting for sex and measuring both males and females will be incredibly important for understanding future population dynamics and potential range expansions.

A future direction that we suggest investigating is whether different developmental stages (e.g. egg, larval, pupal, adult) demonstrate similar responses to temperature. To date, we know of only one study looking at WCR thermal tolerance of the egg stage, which found super cooling points (SCP) between -21 to -27 °C [64]. These SCP values are much lower than reported soil temperatures for cold locations like eastern South Dakota (data from USDA-ARS weather station in Brookings, SD). We are not aware of any study looking at thermal tolerance of WCR larvae, but this might be one of the most abiotically vulnerable life stages for insects as their cuticle is shed during ecdysis leaving new, weaker integument exposed until it has hardened. WCR larvae also reside in the soil, so we might expect them to have reduced thermal tolerance breadth given they are generally buffered from temperature extremes that occur above ground. Data on thermal limits of WCR pupae are non-existent, as is the case for most insects, since it is logistically challenging to measure thermal limits in this life stage. Yet, previous work on WCR suggests temperatures ≥ 33 °C negatively affect survival, development and emergence from the pupal stage [65]. Exploring how each life stage is similar or distinct will provide a more holistic picture of WCR thermal tolerance and aid predictions on how they might move into novel environments.

## Conclusions

Are WCR thermal traits plastic or more constrained? The answer appears to be both yes and no. Certain aspects of an insect’s thermal biology like heat tolerance may be phylogenetically constrained not only across species [12], but also within a species from different geographic origins. In contrast, cold tolerance is often plastic [20], creating a challenge for uncovering similar geographic correlations when working with insects that have been reared in the laboratory for multiple generations. Is WCR then able to leverage the observed differences in heat tolerance across populations? The answer is complicated as WCR is fairly reliant on the presence of their host plant—corn. In the United States, corn is annually planted in more than 80 million acres across 41 states but is grown primarily in the Midwest (e.g. Iowa, Illinois, Nebraska, Minnesota, Indiana, South Dakota; National Agricultural Statistics Service) due to optimal growing conditions including cooler temperatures and wetter summers. However, if corn production continues to expand into warmer locations, then WCR populations from lower latitudes with higher thermal limits will undoubtedly aid this pest species’ expansion.

## Supplementary Information

Supplementary Information.

## Data Availability

All data are publicly available at USDA’s Ag Data Commons or can be requested by contacting the corresponding author, Karl Roeder.
